# A Large Scale Analysis of Information-Theoretic Network Complexity Measures Using Chemical Structures

**DOI:** 10.1371/journal.pone.0008057

**Published:** 2009-12-15

**Authors:** Matthias Dehmer, Nicola Barbarini, Kurt Varmuza, Armin Graber

**Affiliations:** 1 Institute for Bioinformatics and Translational Research, UMIT, Hall in Tyrol, Austria; 2 Department of Computer Engineering and Systems Science, University of Pavia, Pavia, Italy; 3 Institute of Chemical Engineering, Laboratory for Chemometrics, Vienna University of Technology, Vienna, Austria; University of California, Berkeley, United States of America

## Abstract

This paper aims to investigate information-theoretic network complexity measures which have already been intensely used in mathematical- and medicinal chemistry including drug design. Numerous such measures have been developed so far but many of them lack a meaningful interpretation, e.g., we want to examine which kind of structural information they detect. Therefore, our main contribution is to shed light on the relatedness between some selected information measures for graphs by performing a large scale analysis using chemical networks. Starting from several sets containing real and synthetic chemical structures represented by graphs, we study the relatedness between a classical (partition-based) complexity measure called the topological information content of a graph and some others inferred by a different paradigm leading to partition-independent measures. Moreover, we evaluate the uniqueness of network complexity measures numerically. Generally, a high uniqueness is an important and desirable property when designing novel topological descriptors having the potential to be applied to large chemical databases.

## Introduction

The problem to quantify the complexity of a network appears in various scientific disciplines [1]–[7] and has been a challenging research topic of ongoing interest for several decades [8]. This problem first appeared when studying the complexity of biological and chemical systems, e.g., battery cells or living systems [9]–[12] using information-theoretic measures [13] (in this paper, we use the words “measure”, “index”, “descriptor” synonymously when referring to topological graph complexity measures). Directly afterwards, the idea of applying entropy measures to network-based systems finally emerged as a new branch in mathematical complexity science. An important problem within this area deals with determining the so-called structural information content [8], [12], [14]–[19] of a network. Finally, it turned out that the developed information indices for measuring the information content of a graph have been of substantial impact when solving QSPR (Quantitative structure-property relationship)/QSAR (Quantitative structure-activity relationship) problems in mathematical chemistry and drug design [1], [2], [20]–[25]. Correspondingly, such measures have been widely used to predict biological activities as well as toxicological and physico-chemical properties of molecules using chemical datasets, see, e.g., [1], [20], [23]–[26]. More precisely, most powerful and generally applicable for theses approaches are empirical multivariate models 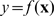, with 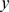 being a chemical or a physical property (P) or a biological activity (A), and vector 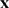 consisting of a series of numerical molecular descriptors describing the molecular structure. For modeling biological activities also (measured or computed) physical properties are used. Some of the already mentioned information-theoretic complexity measures which are well-established in mathematical chemistry will be defined in the next section.

Before sketching the aims of our paper, we start with a brief review about classical and more recent approaches to measure the complexity of networks. However, for performing the numerical results, we mainly restrict our analysis to information-theoretic measures which are based on Shannon's entropy [13] and which have already been applied in the context of mathematical chemistry [2], [21] and drug design [1], [20], [23].

In general, it seems clear that *complexity* and, even, *structural complexity* is generally not uniquely defined because it is in the eye of a beholder [27]. Consequently, it is often not clear which structural features of a graph in question should be taken into account. For instance, to use complexity measures within mathematical chemistry, some of their desirable features were stated in [3]. Now, we start outlining the most known classical approaches and then turn to more recently developed approaches for detecting network complexity. Beside the already mentioned information-based measures [1], [2], [8], [20]–[26], [28], the complexity of a network was also defined by using boolean functions approaches [6], [8], [29], [30]. For example, Constantine [29] defined the complexity of a graph to be the number of its containing spanning trees. Jukna [30] determined graph complexity as the minimum number of union and intersection operations required to obtain the whole set of its edges starting from star graphs. Finally, the so-called combinatorial complexity of a network was developed by Minoli [6]. The key property of such a descriptor is that it must be a monotonically increasing function of the factors which contribute to the complexity of a network, e.g., number of vertices and edges, vertex degrees (branching [3]), multiple edges, cycles, loops, and labels [3]. Another crucial definition of complexity (algorithmic information) that is different compared to the mentioned ones was given by Kolmogorov [31]. Based on appropriate string encodings of graphs, bounds to estimate the Kolmogorov-complexity of labeled and unlabeled graphs were obtained in [32]. However, this kind of network complexity measures are difficult to apply in general because of computational reasons [32]. In order to briefly review more recently developed approaches, we start by mentioning some quantities for structurally characterizing networks [33], [34] which emerged from complex network theory [33], [35]–[37]:

- Size of the giant connected component [33], [38].
- Degree distributions 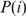 [33], [38]–[41].
- Exponent of degree distributions [33], i.e., it holds 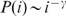.
- Total number of vertices and edges [33], [34], [40], [42], [43].
- Path-based quantities [33], [40], [42], [44].
- Distance-based quantities, e.g., 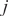-spheres, average distances, eccentricity, diameter and radius [33], [40], [42], [44].
- Degree, degree statistics and edge density [33], [40], [42], [44].
- Clustering coefficient, modularity and network motifs [45]–[48].
- Eigenvector measures [40], [49], [50].

Further, various measures have been developed to characterize the complexity of networks where many of the recent ones were summarized by Kim et al. [51] and da Costa et al. [44]. In particular, information-theoretic complexity measures for general graphs have been investigated in [51]–[54]. For instance, starting from directed networks, the information measure called Medium Articulation was defined which is maximized for exactly the medium number of links [53]. Properties thereof were examined in [54]. Another entropy-based measure called Offdiagonal complexity (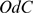) was contributed by Claussen [52]. This graph complexity measure is based on determining the entropy of the so-called offdiagonal elements of the vertex-vertex link correlation matrix [51], [52]. Similar entropy measures can be also found in [44], [55]. We already mentioned that the number of spanning trees might also serve as graph complexity measure, see, e.g. [29]. As a further attempt, Kim et al. [51] developed a more sophisticated approach by calculating a quantity for each edge that takes the number of spanning trees of the graph and the number of spanning trees of the corresponding one-edge-deleted subgraph into account. By using these entities which were called sensitivities, an entropic measure was defined and interpreted as a spanning tree sensitivity complexity of a network. Another important class of network complexity measures is based on determining subgraphs of a network [51], [56]. More precisely, the concrete idea is as follows: The more different subgraphs a network contains, the more complex is the underlying network [51]. Here, “different” means that non-isomorphic graphs are considered, however, the graph isomorphism problem is known to be computationally costly, see, e.g. [57], [58]. Thus, Kim et al. [51] proposed approximations for decide graph isomorphism and ended up with several subgraph-based graph complexity measures which can be found in [51]. Further, methods based on characterizing subgraph relationships were developed in [56]. To finalize our review on general graph complexity measures, we mention two recently developed approaches [59], [60]. In [59], measures were proposed capturing features around each vertex to identify singular vertices. As an interesting result, they found that the obtained singular motifs had unique functional roles in the considered network [59]. A statistical method was defined in [60] to detect network regularity interpreted as simplicity. Finally, starting from a set of measurements and by applying PCA analysis, they found simple regions in the networks under consideration [60]. Interestingly, we want to point out that these two approaches are particularly interesting for investigating biological networks (but not limited to). Especially, the latter method takes incompleteness or noise during the network construction into account [60]. However, the chemical graphs we will use in our paper are deterministically inferrable and not erroneous (measurement errors). This is the reason why we restrict our analysis to information-theoretic measures for globally quantifying the information content of chemical structures where the probability distribution is deterministically inferrable from structural features (e.g., orbits and 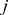-spheres) of the graphs in question.

In this paper, we investigate information-theoretic network complexity measures which are particularly relevant for enhancing empirical QSAR/QSPR models [23]. As we have already expressed, a variety of graph measures have been used so far to characterize the so-called molecular complexity [3], [6], [61], [62]. However, many of such complexity measures lack a meaningful interpretation. Thus, as the major contribution of our paper, we put the emphasis on examining interrelations between information-theoretic network complexity measures often used in mathematical chemistry, that is, we shed light on the problem which kind of structural information the measures detect when applied to chemical graphs.

To tackle this problem, we select a few measures from two different paradigms for inferring such indices: The so-called topological information content [19] (see Equation (4) of a graph and information measures (see Equation (23)) based on using special information functionals [63]–[65]. The former represents a classical *partition-based* measure that relies on symmetry with respect to topologically equivalent vertices having the same degrees. The latter is a *partition-independent* information measure that is based on using a special information functional capturing structural features of the networks. In order to perform this study, we evaluate these measures numerically by using several large datasets containing real and synthetic chemical graphs. To our best knowledge, such a large scale analysis involving the classical topological information content has not been done so far. Note that in this study, we only consider skeletons of the chemical structures, that is, all atoms are equal and all bonds are equal. Another problem we want to address in this paper is to investigate the uniqueness of complexity measures. This relates to examine their discrimination power, that means, their ability to discriminate non-isomorphic graphs as unique as possible. For this, we also use the mentioned databases - real and synthetic chemical structures - and calculate a special sensitivity measure [66]. Besides evaluating the uniqueness of the information-theoretic measure introduced in the next section, we will calculate the sensitivity values of the entropic measure Offdiagonal complexity and the graph index (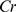 is a non-information-theoretic graph complexity measure) 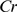, see [51]. Finally, our research addresses the challenging problem of investigating the capability of information-theoretic network descriptors for meaningfully capturing structural features of graphs.

## Methods

This section aims to present the information-theoretic topological descriptors we want to investigate in this paper. In the following, we briefly shed light on the two main procedures (resulting in partition-based and partition-independent measures) to infer information-theoretic complexity measures for characterizing chemical network structures. Afterwards, we express their concrete definitions for performing our numerical analysis.

### Information-Theoretic Network Complexity Measures

Applying information-theoretic methods for exploring complex networks is a still challenging and ongoing problem [7]–[9], [12], [14], [15], [19], [55], [67], [68]. As mentioned in the introduction, this research area has its origin in biology and mathematical chemistry [8], [9], [12], [19]. Historically seen, Trucco [12] and Rashevsky [19] were the first who developed information measures to analyze complex biological and chemical systems. Later, Mowshowitz [15]–[18] further developed this approach and proved important mathematical properties thereof.

More precisely, Trucco [12] and Rashevsky [19] defined entropy measures for graphs which were interpreted as the structural information content of a graph; the original information measure due to Rashevsky [19] is called the so-called topological information of a graph in question, see Equation (4). So far, the just mentioned information measures representing the entropy of the underlying graph topology have been widely used for measuring the structural complexity of graphs [3], [15]–[18], [21], [27], [55], [69]. The basic principle to infer these measures is as follows: Let 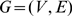 be a graph. By starting from an arbitrary graph invariant 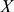 of 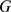 and an equivalence criterion 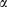, one obtains a partitioning of 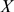 where the partitions are denoted by 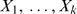. In order to infer probabilities for each obtained partition, the entities 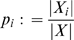 can be used because it obviously holds
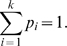
(1)Thus 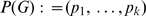 represents a finite probability distribution of 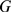. Now, applying Shannon's entropy formulas [13] leads to the classical graph entropies [8]:
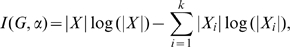
(2)

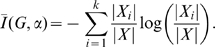
(3)


Equation (2) is the total information content of 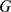, whereas Equation (3) represents its mean information [2], [70]. We want to point out that the just explained procedure yields to partition-based information measures for determining the structural complexity of networks. For example, Mowshowitz [15] obtained such a measure based on algebraic equivalence criteria, e.g., graph automorphisms and graph colorings [15], [57]. But it is known that the problem of determining graph automorphisms is equivalent to check whether two graphs are isomorphic [71]. Moreover, the computation of the chromatic number of undirected graphs to infer chromatic decompositions was proven to be NP-complete [58]. Hence, one can expect that the computational complexity of the underlying algorithms for calculating these measures are for arbitrary graphs very costly. After this seminal work [15]–[19], the outlined principle of inducing vertex partitions was generalized by associating a weighted finite probability distribution to a network, see [8]. This generalization led to numerous information-theoretic graph complexity measures by applying equivalence criteria like vertex degrees, distances to chemical graphs etc. [2], [8], [21].

Now, we give a sketch of the second procedure for inferring graph entropy measures that results in obtaining partition-independent measures [63]–[65]. The main idea is as follows: Instead of inducing vertex partitions to obtain probabilities for subsets of vertices, we assign a probability value to every vertex in a graph. This has been done by means of so-called information functionals [64], [65] (note that concrete information functionals will be defined in the next section) which capture structural features of a graph and here represent positive mappings which are assumed to be monotonous, see, e.g., [63]. A notable feature of this procedure is that we avoid the problem of determining vertex partitions associated with an equivalence relation that can be often computationally expensive.

As follows, we start with the definition of some concrete partition-based entropy measures to be applied to real and synthetic chemical structures. Note that in this paper, we only evaluate the mean information contents. For the sake of simplicity, we write 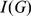 instead of 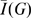.

#### Definition 1

*Let* 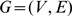 *be a graph.*

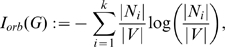
(4)
*is called topological information content of* 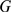 *. Here,* 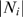 *denotes the number of topologically equivalent vertices in the* 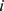 *-th vertex orbit of* 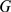 *where* 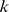 *is the number of different orbits.*

#### Remark 1

*Let* 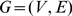 *be a graph. We recall the definition* [*2*] *for two vertices* 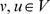 *being topologically equivalent: For each* 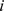 *-th neighboring vertex of* 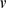 *there exists an* 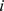 *-th neighboring vertex of* 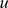 *which possesses the same degree. A vertex orbit is a set of vertices that only contains topologically equivalent vertices.*

#### Definition 2

*Let* 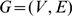 *be a graph.*

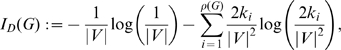
(5)

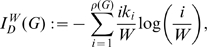
(6)
*where*

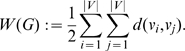
(7)

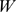
 *is called the Wiener index* [*72*] *and* 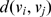 *denotes the shortest distance between* 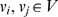 *.* 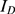 *and* 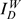 *are so-called magnitude-based information indices, see* [*69*] *. It is assumed that the distance of a value* 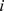 *in the distance matrix* 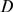 *appears* 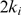 *times.* 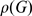 *stands for the diameter of a graph* 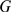 *.*

#### Definition 3

*Let* 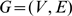 *be a graph.*

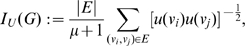
(8)

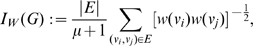
(9)
*where*

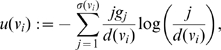
(10)

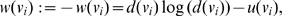
(11)

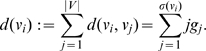
(12)
*See* [*28*] *.* 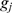 *equals the number of vertices having distance* 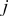 *starting from* 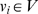 *. Also,* 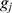 *equals the corresponding* 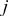 *-sphere cardinality.* 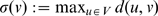 *is the eccentricity of* 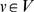 *.* 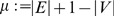 *denotes the cyclomatic number, see* [*28*] *.*

#### Definition 4

*Let* 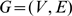 *be a graph.*

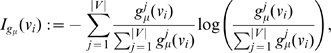
(13)
*where*

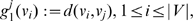
(14)

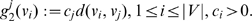
(15)

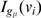
 *is a local vertex entropy* [*66*] *. Finally, the entropy of* 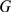 *can be defined by*

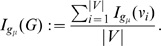
(16)


In particular, we define special information measures for characterizing graphs by choosing concrete coefficients [73].

#### Definition 5

*Let* 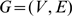 *be a graph. We define*

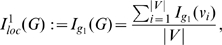
(17)

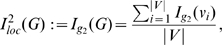
(18)
*where*

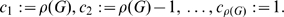
(19)
*Finally,*

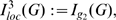
(20)
*where*

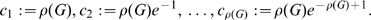
(21)


To finalize this section, we now express the definitions of some partition-independent entropy measures for graphs introduced by Dehmer et al. [63]–[65]. Mathematical properties and applications thereof can be found, e.g., in [64], [65].

#### Definition 6

*Let* 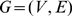 *be a graph. The following partition-independent entropy measures based on a special information functional were defined as* [*63*], [*65*]

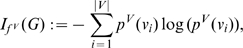
(22)

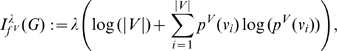
(23)
*where* 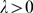 *is a scaling constant.*

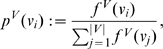
(24)
*are vertex probabilities. The special information functional* 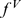 *was defined as* [*63*]

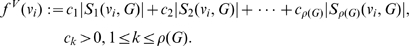
(25)
*Here,* 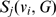 *denotes the* 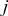 *-sphere* [*65*] *of a vertex* 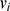 *, that is, the set of vertices having shortest distance* 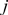 *starting from* 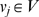 *.* 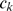 *are positive coefficients for emphasizing certain structural of a graph, e.g., high vertex degrees, also see,* [*63]*, [*65*] *.*

#### Remark 2

*To perform the numerical calculations in this paper, we set* 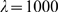 *.*

#### Definition 7

*Let* 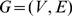 *be a graph. The measure* 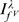 *becomes to* 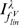 *by choosing the coefficients* 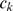 *according to* *Equation (19* *), i.e., linearly decreasing. Correspondingly,* 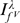 *becomes to* 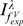 *when choosing the coefficients* 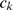 *according to* *Equation (21* *), i.e., exponentially decreasing.*

In the following, we briefly comment on the computational complexity of the discussed information measures without giving proofs. Obviously, the measures whose definitions are based on calculating matrices can be often computed in polynomial time (e.g., square, cubic etc.). For instance, it has been proven [74] that the fastest general algorithm to compute the Wiener index is 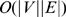. Applying 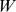 to trees, its computation even only requires time complexity 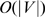. To calculate 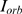, the automorphism group of the corresponding graph has to be formally determined. However, it is well known that this procedure is computationally extensive for arbitrary graphs [71]. Hence, this measure is rather not suitable to calculate the information content of large networks. If 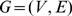 is an undirected and connected graph, we showed in [65] that the computation of 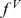 requires time complexity 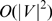. By applying a shortest path algorithm 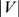 times, it easily follows 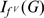 has time complexity 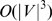. In order to examine the time complexity of such indices which are based on determining shortest paths for every vertex in a graph, e.g., 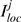, one can argue almost analogously. Further, it can be similarly shown that the remaining information measures possess polynomial time complexity. The computational complexity of 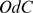 and 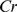 (see next section) has already been discussed in [51], [52].

### Additional Network Complexity Measures

As stated in the introduction, we will additionally evaluate the uniqueness of the Offdiagonal complexity and the graph index 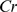, see [51], [52].

#### Definition 8

*Let* 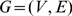 *be a graph and let* 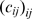 *be the vertex-vertex link correlation matrix, see* [*52*] *.* 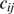 *denotes the number of all neighbors with degree* 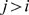 *of all vertices with degree* 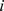 ** [*51*] *.* 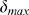 *stands for the maximum degree of* 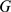 *. The normalized version of* 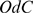 *can be defined as* [*51*]

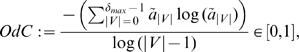
(26)
*where*

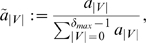
(27)
*and*

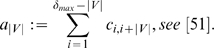
(28)


#### Definition 9

*Let* 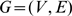 *be a graph and let* 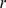 *be the largest eigenvalue computed from its adjacency matrix.*

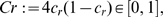
(29)
*where*

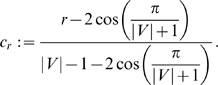
(30)


Before discussing numerical results, we describe the databases and our developed software in brief.

### Chemical Graph Databases

- MS 2265: This database has been extracted by own software from the commercially available mass spectral database NIST [75]. It contains 2265 selected chemical structures with different skeletons originating from the database NIST. This database has been already used in [63] for investigating different aspects of topological descriptors. It holds 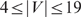; 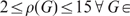 MS 2265.
- AG 3982: The original freely available database called Ames Genetoxicity contains 6512 chemical compounds, see [76], [77]. After filtering the isomorphic graphs by using SubMat [78], we obtained 3982 structurally different skeletons, that is, all atoms and all bonds are considered as equal. The database was created from six different public sources [76], [77]. Each structure has a class label (0 and 1) that results from the so-called Ames test indicating the genetoxicity of a substance. So far, the mentioned test has often been used in pharmaceutical sciences when investigating new molecules [76]. It holds 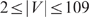; 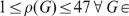 AG 3982.
- APL 91075: The ASINEX Platinum Collection is a freely available, in-house designed and synthesized collection of 126615 drug-like compounds [79], [80]. The filtering process of the isomorphic graphs by using a Python program resulted in 91075 structurally different skeletons. A notable feature of this database is that it contains structures from chemical subareas which are often under-represented in other available structure libraries [80]. Here, the chemical structures represent unlabeled and undirected graphs (skeletons). It holds 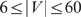; 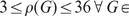 APL 91075.
- C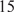 trees: This synthetic graph class [63] consists of 4347 alkane isomers with 15 carbon atoms (vertices). By definition, trees are connected, cycle free and here represent unlabeled and undirected graphs (skeletons). This database has been created by the software Molgen, see also [63].
- C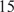 ring 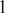: This synthetic graph class [63] consists of 60077 hydrocarbon isomers with 15 carbon atoms (vertices) containing one ring 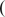
cycle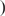 and only single bonds. Hence, the structures can be treated as unlabeled and undirected graphs (skeletons). This database has been created by the software Molgen, see also [63].
- C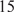 ring 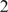: This synthetic graph class [63] consists of 94013 hydrocarbon isomers with 15 carbon atoms (vertices) containing two rings 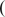
cycles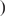 and only single bonds. Hence, the structures can be treated as unlabeled and undirected graphs (skeletons). This database has been created by the software Molgen, see also [63].

### Software and Data Processing

In order to generate and process our chemical graphs, we used the known Molfile format [81]. The database AG 3982 was originally available in Smiles format that we converted to Molfile format (SDF) using a Python procedure. The databases MS 2265 and APL 91075 were directly available in Molfile format (SDF). To apply the information-theoretic measures to the previously presented graph databases, we performed a procedure to filter all isomorphic graphs contained in these databases. This isomorphism check was done by applying the software SubMat [78] and the previously mentioned Python program. As a result, we obtained sets of graphs containing different skeletons representing the underlying graph topology of the molecules.

We implemented all used topological measures in Python using freely available libraries like Networkx, Openbabel and Pybel packages [82]. For the calculations we have performed in this paper, we started from the Molfile representation of a chemical structure, created the corresponding adjacency matrix and computed the topological indices based on the developed Python program. The databases containing the synthetic graph structures (isomers) have been generated by the software Molgen, see also [63].

## Results and Discussion

In this section, we will apply the complexity measures presented in the previous section. As stated before, we mainly put the emphasis on exploring the relatedness between the topological information content 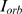 and our graph entropy measures 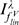 and 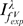. Moreover, we numerically calculate further information-theoretic network measures presented in the last section and interpret the results. In particular, an interesting question will be to investigate the so-called uniqueness of the measures when applying them to both databases containing real and synthetic chemical graphs.

### Numerical Results

In the following, we discuss and interpret numerical results when applying the selected descriptors to sets containing real chemical structures. Our study involves calculating and interpreting dependency plots, cumulative entropy distributions, and the so-called uniqueness of the used topological indices [66].

#### Relatedness between 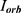 and 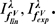


**Figure 1:**
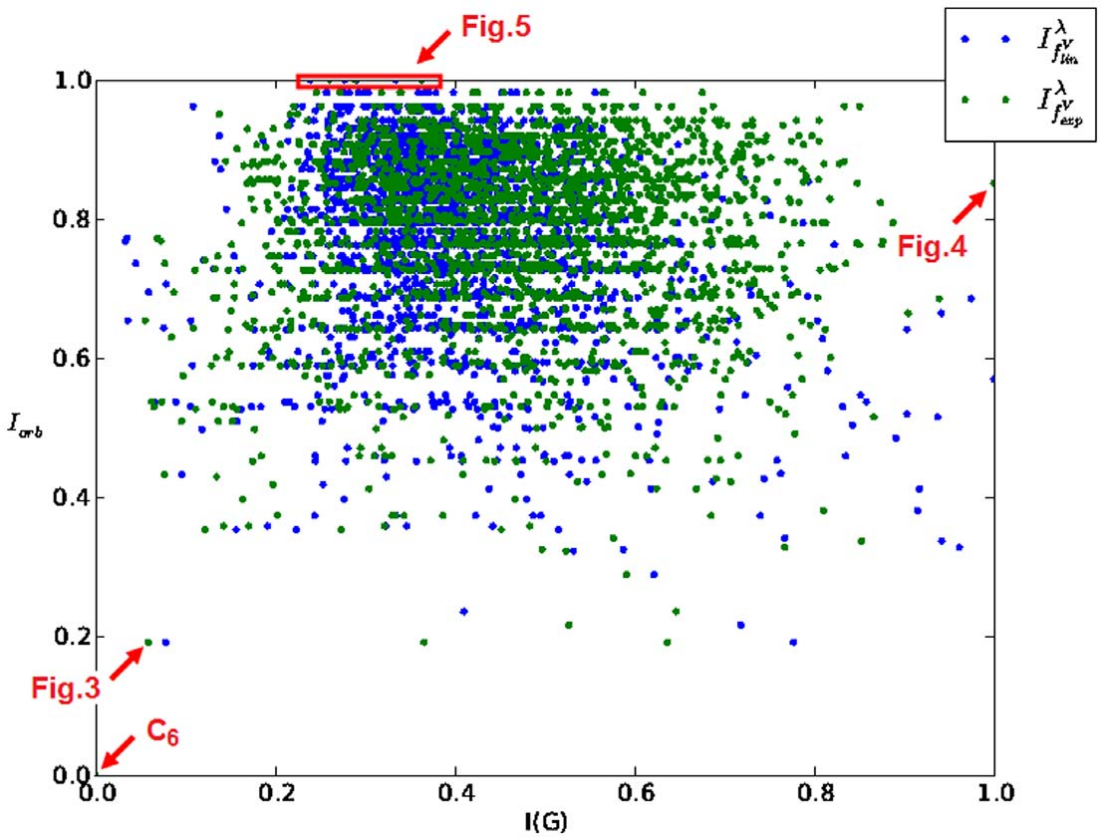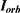 versus 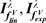 for MS 2265. (reference label: scatter_plot1).

**Figure 2:**
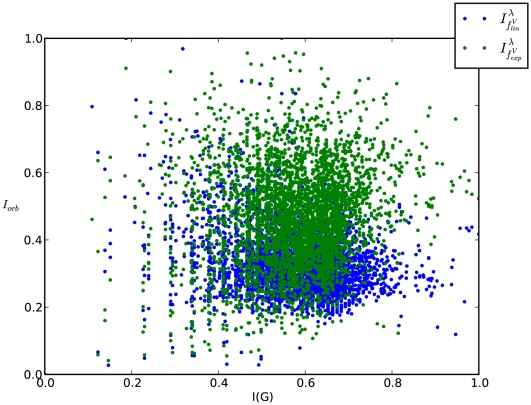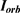 versus 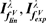 for AG 3982. (reference label: scatter_plot2).

**Figure 3:**
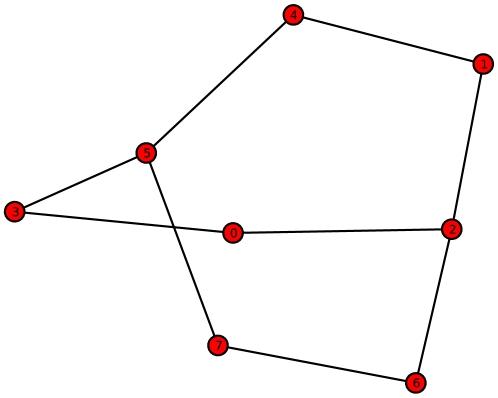
Example Graph with relatively small value of both 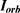 and 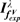. (reference label: graph_plot1).

**Figure 4:**
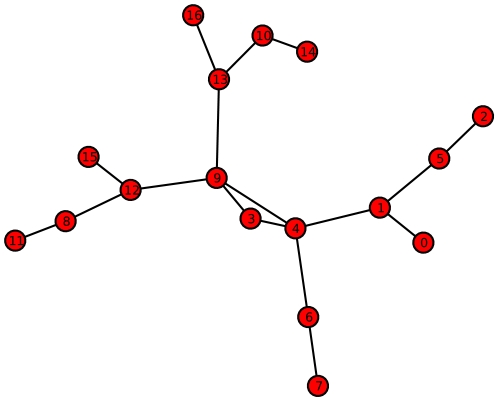
Example Graph 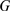 with relatively large value of 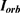 and 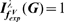. (reference label: graph_plot2).

**Figure 5:**
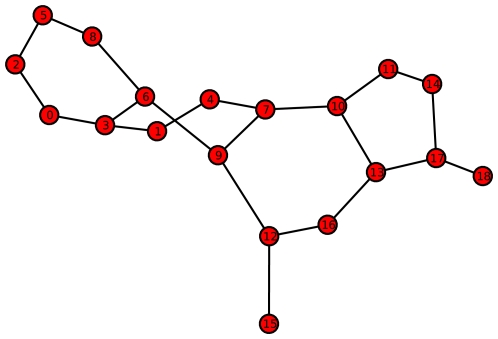
Example Graph 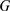 with 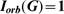 and relatively small 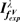. (reference label: graph_plot3).

We start to examine how the entropies 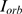 and 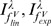 capture structural information of our graphs and depict the scatter plots (see Figure (1) and Figure (2)) for exploring the correlation between the measures. To tackle this problem, we now only consider Figure (1) exemplarily. Clearly, the main observation is that 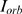 is highly uncorrelated with 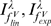. In order to interpret this figure in more detail, we select the graphs marked by red-colored arrows (these graphs are depicted in Figure (3), (4), (5)) whose entropies (for practical scaling reasons, we always calculated normalized entropies) are extremal with respect to 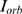 or 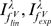. Before discussing the results, we give two mathematical statements [27], [64].

##### *Proposition 1*

*If* 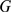 *is vertex transitive* [*27]*, [57**] *, then* 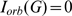


##### *Proposition 2*

*If* 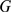 *is* 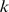 *-regular* [*57*] *, then* 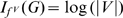 *and, hence,* 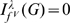 *.*

The graph 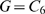 with 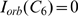 and 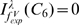 is a cycle possessing six vertices (Figure (1)). Because 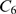 is vertex transitive, there is only one orbit containing all vertices and, thus, according to Proposition (1), we get 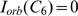. Moreover, 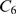 is 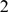-regular. Applying Proposition (2) yields to 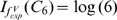 (see also Equation (22)) and, hence, 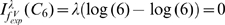.

The interrelation between the entropies (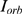 and 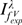) for the graph depicted by Figure (3) can be understood by applying the previously stated propositions. As we easily see, this graph has a cyclic and symmetric structure and, therefore, 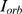 is low. For the same reason when explaining the interrelation for the fully cyclic graph 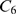, the corresponding entropy value of 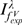 is also low. The next entropy relation we want to describe concerns the graph 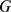 (see Figure (4)) whose topological information content is relatively high and 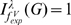. Here, 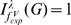 means that the entropy 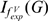 attains a minimum. The reason why the topological information content is relatively high for this graph can be understood by the fact that the degree of symmetry is rather low resulting in the observation that most of the vertex orbits of 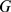 are only singleton partitions. The last graph 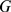 we will inspect possesses 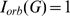 and a relatively small value of 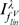. This graph 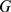 (see Figure (5)) is an element of a certain subset that is highlighted by the red-colored rectangle in Figure (1). To determine 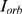 for this graph, we have to calculate the partitions according to the equivalence criterion that is based on vertex orbits. At first glance, 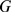 seems to be symmetric (according to this criterion) but a deeper inspection leads to the result that all vertex orbits are singleton partitions. Hence, 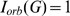. But based on the cyclic structure of 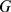 and again by definition of 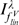 and Proposition (2), we infer that its corresponding entropy value is relatively small.

#### Uniqueness of the Descriptors

Besides investigating the problem how the measures capture structural information of the considered chemical structures, we now examine another important property of a topological index, namely the ability to discriminate the graphs as unique as possible. This characteristic property of a structural graph measure is often referred to as degeneracy [66], [69], [83]; related work can be found in, e.g., [63], [66], [69], [83], [84]. To evaluate the uniqueness of a measure 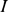, we here apply the sensitivity index proposed by Konstantinova [66]:
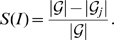
(31)



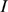
 denotes a topological index and 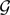 denotes a set of arbitrary graphs, respectively. 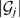 stands for the number of graphs 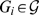 which can not be distinguished by calculating 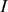. If it holds 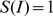, we know by definition that it does not exist any pair of non-isomorphic graphs 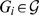 possessing the same value of 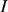.

We now start discussing the results shown in Table (1) when evaluating the sensitivity of our indices and start with the topological information content 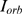. We note that the sensitivity values depends on the chosen decimal places. Here, we calculated 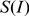 with an accuracy of 6 decimal places. First, we see that 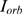 has a very low discrimination power compared to the remaining information measures, except 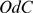 and Wiener index. This can be understood by briefly recalling the definition of the topological information content (see also Remark(1)): The main idea is to partition the vertex set in equivalence classes according to the criterion that each such class contains topologically equivalent vertices [2], [19]. Therefore, this measure is based on symmetry with respect to the topologically equivalent vertices having the same degrees (the vertices to be in the same vertex orbit must have the same degree). Thus, we can easily construct graphs having the same vertex orbits but whose underlying topology is different, and, evidently, the uniqueness of 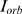 is often very low. Interestingly, 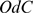 has similarly to 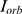 a very low discrimination power. This can be explained by arguing that the underlying basis for calculating this measure - the vertex-vertex link correlation matrix - does not capture complex structural features of a graph adequately (at least for the considered graph classes). As known and reflected by Table (1), the uniqueness of the Wiener index is also very low [66]. In contrast to this, the sensitivity values of 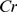 for MS 2265 and AG 3982 are feasible. But for APL 91075, its uniqueness is very low. This clearly shows that the uniqueness of a topological index strongly depends on the graph class (structural diversity of graphs) under consideration (see also “Summary and Conclusion” section). Note that the sensitivity calculation of our information indices 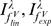 led to much better results. By choosing the coefficients exponentially decreasing (see Equation (21)), the resulting entropy measure is able to discriminate all graphs of MS 2265 uniquely and, hence, 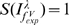. For AG 3982 and APL 91075, we obtained that 12 and 220 graphs could not be distinguished when applying 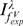, respectively. The sensitivity evaluation of 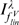 led to quite similar result. In summary, Table (1) shows that our information indices possess a very high uniqueness for all three chemical databases and, therefore, can discriminate real chemical graphs successfully. A more mathematical explanation for this result is as follows: Instead of determining partitions by using a graph invariant, e.g., number of vertices or edges, and then calculating a probability for each such partition, we assign a probability value to every vertex in a graph. By using our proposed information functional, we furthermore compute the full topological neighborhood of all involved vertices (atoms) of the structure [63]. To determine the entropy of the underlying graph topology, the vertex probabilities (see Equation (24)) can be interpreted as percentage rates of the entire graph structure for every vertex instead of lumping structural properties together when calculating the partitions (according to a an equivalence criterion). As a conclusive remark, we want to emphasize that 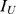 and some other computed information indices also possess a high discrimination power (see Table (1)).

**Table 1:** Calculation of sensitivity index 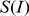 for chemical databases.

Topological index 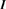	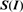 for MS 2265	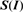 for AG 3982	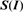 for APL 91075
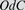	0.142604	0.247363	0.029744
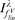	0.997350	0.995981	0.988723
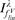	1.0	0.996986	0.997584
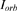	0.026931	0.074334	0.002723
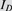	0.859602	0.938724	0.873873
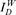	0.883885	0.947513	0.933033
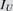	0.999116	0.999497	0.996618
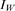	0.990286	0.990959	0.522799
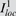	0.995584	0.994977	0.914389
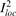	0.999116	0.996986	0.916453
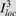	0.989403	0.973882	0.595783
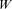	0.014128	0.037920	0.001065
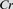	0.864017	0.919638	0.223892

To interpret the sensitivity values when applying our information measures to synthetical chemical graphs, we look at Table (2). Here, we applied the same graph measures to the presented synthetic graph classes. As before, the uniqueness of 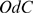, 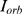 and 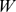 is for all three graph classes extremely low. Compared to 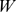, one sees that 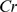 has a much better discrimination power. By exemplarily determining the number of graphs which could not be distinguished by 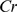 for C15 ring 2, we yield 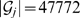 (see Equation (31)).

**Table 2:** Calculation of sensitivity index 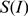 for synthetic graph classes.

Topological index 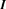	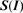 for C15 trees	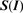 for C15 ring 1	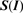 for C15 ring 2
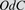	0.001380	0.000065	0.000031
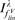	0.983897	0.963713	0.980034
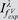	1.0	0.998601	0.997383
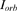	0.001380	0.000116	0.000042
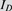	0.634000	0.124972	0.093774
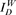	0.748562	0.142567	0.108889
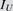	0.998159	0.937213	0.859530
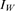	0.987577	0.771842	0.586365
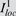	0.965263	0.568736	0.394817
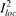	0.965033	0.669940	0.553370
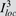	0.982286	0.785658	0.727622
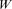	0.000920	0.000116	0.000085
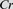	0.459627	0.502771	0.491857

However for the tree class, our 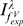 discriminates all 4347 trees uniquely. Moreover, one observes that the sensitivity values of the remaining information measures for this graph class are high. The final result we want to emphasize is that by applying our information-based topological descriptors 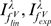, we obtained constantly high sensitivity values for all three synthetic graphs classes. In order to calculate the number of graphs which could not be distinguished by 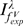 and 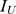, we choose again the class C15 ring 2. For 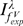, we get 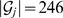 but by applying 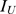, we yield 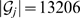.

#### Cumulative Entropy Distributions

**Figure 6:**
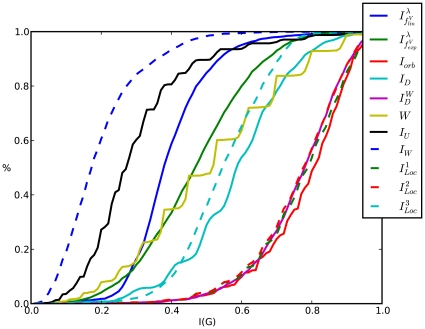
Cumulative Entropy Distributions for MS 2265. (reference label: cum_plot1).

The cumulative entropy distributions are illustrated by Figure (6). In these plots, the 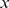-axis represents the normalized entropy values whereas the 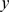-axis shows the percentage rate of chemical graphs having a (normalized) entropy value less or equal 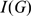. We want to remark that the measures were normalized by using 
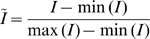
.

We start by observing that about 80% of the graphs of MS 2265 possess relatively small entropy values when evaluating 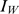 (see Equation (9)). In contrast, 80% of the graphs have large entropy values by calculating 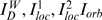 (see Equation (6), (17), (4)). This result can be interpreted such that the measures capture structural information of the graphs quite differently because the corresponding entropy distributions are almost reverse. The interrelation between the graph entropies 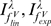 (see Equation (23)) and 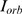 is quite similar to the just described one. Finally, note that the findings of the section where we have examined the relatedness between the selected measures support this hypothesis.

**Figure 7:**
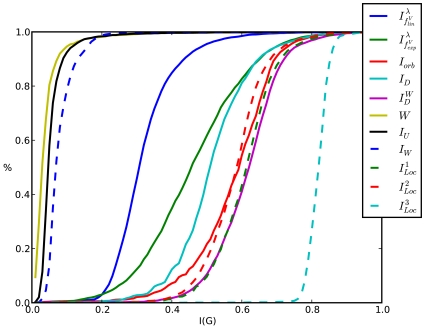
Cumulative Entropy Distributions for AG 3982. (reference label: cum_plot2).

**Figure 8:**
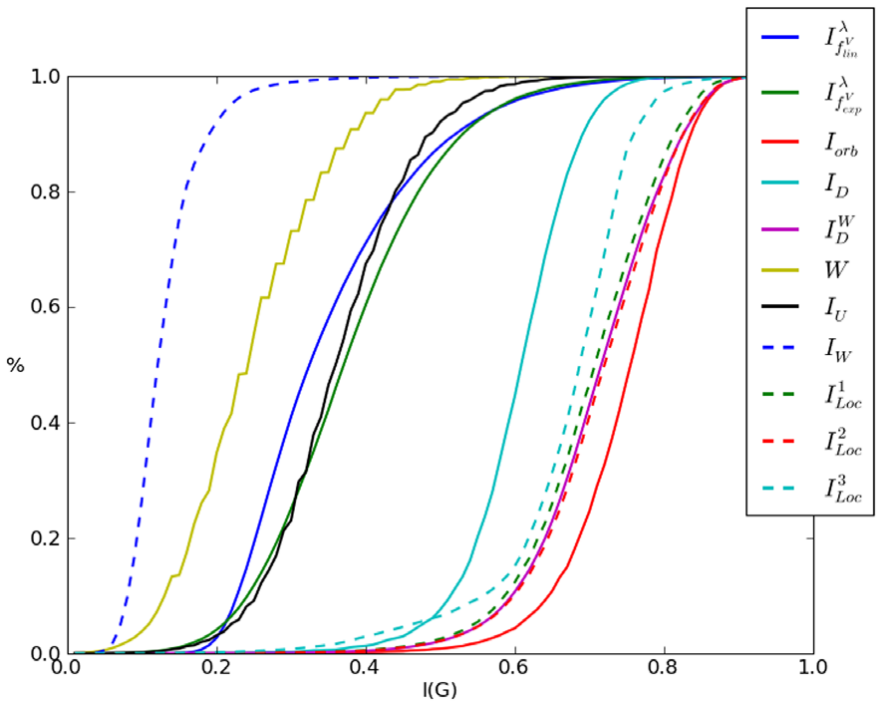
Cumulative Entropy Distribution for APL 91075. (reference label: cum_plot3).

Equally, the cumulative entropy distributions of AG 3982 are depicted in Figure (7). One can see that for some indices the curve progressions appear quite diversely, e.g., 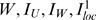. A possible explanation for this could be the fact that AG 3982 is structurally more diverse than MS 2265. For the remaining entropy measures, the situation is similar as described in Figure (6). Interestingly, the cumulative similarity distribution of the discussed information measures illustrated by Figure (6) and Figure (8) are again quite similar.

In particular, we have found that for all three chemical databases, the evaluation of the topological information content (see Equation (4)) and the partition-independent measures (see Equation (23)) led to clearly different cumulative entropy distributions that is obviously in accordance with the results of the preceding sections.

### Summary and Conclusion

In the present paper, we studied interrelations between classical and novel entropy measures to quantify the structural information content of networks. Here, these measures served as graph complexity measures which take certain structural features of the networks under consideration into account. In the following, we express the main findings of the paper in brief:

- We explored the relatedness between information measures for graphs. In particular, we examined the correlation between the topological information content 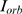 (see Equation (4)) and the partition-independent measures 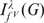 (see Equation (23)) by interpreting the corresponding scatter plots. Let 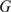 be a graph. If 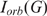 is small or even zero, then 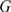 is symmetric with respect to topologically equivalent vertices having the same degrees which form the so-called vertex orbits. Then, if the value of 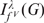 is also small, 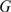 has a cyclic structure and represents a graphs that is equal or very similar to a 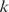-regular graph. As shown in Figure (5), a graph 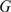 whose value of 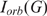 is large can be also cyclic and, hence, possesses a small 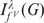 value. Further, for a graph 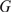 whose value of 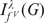 is large (Figure (4)), the involved mean information content 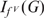 is low or even attains a minimum. In [63], we showed that such graphs typically represent chain-like graphs or generally speaking, graphs with a low branching factor. The reason why 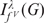 has small values for graphs containing cyclic structures seems (which are symmetric) logical because it corresponds to the accepted concept [3] that symmetry leads to a decrease of complexity.
- Another important aspect of our numerical study was to examine the discrimination power of the used network measures. We found that the topological information content 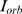 was weak in distinguishing non-isomorphic graphs, i,e., it's sensitivity value was very low. In contrast, the sensitivity evaluation for our partition-independent measures 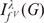 led to constantly good results when applying the measures to real and synthetic chemical structures. Recall that a high uniqueness of a complexity measure corresponds to the ability to distinguish networks whose structural similarity is very high. Hence, this feature could be useful (as future work) when considering graphs which were inferred statistically (erroneous graphs) [85]. As an important remark, we want to emphasize that the uniqueness of a topological index also depends on the considered graphs class. Note that our chemical graphs are particularly small and structurally not very diverse compared to the ones used in e.g., [60]. Especially for those graphs whose numbers of vertices are rather small, highly discriminative measures are extremely important for quantifying structural information as unique as possible. That is one reason why we studied the uniqueness of topological indices for chemical graph analysis. A further reason relates to the fact that descriptors with a high discrimination power are often useful for QSPR/QSAR. But we have already seen that an index 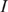 does not necessarily perform well for several graph classes at the same time. To further shed light on this problem, we briefly pick up the first argument of this paragraph. In this paper and in [84], we evaluated the uniqueness of some information-theoretic measures for real and synthetic chemical structures. For some indices, e.g., 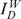, 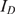 which performed very well for real chemical graphs, we got worse results when applying these measures to synthetic graphs, e.g., isomers having 10 [84] and 15 vertices each.
- For the real chemical databases, the cumulative entropy distributions of some measures were calculated. This approach can be considered as an important preprocessing step to learn how the measures capture structural information of networks. Particularly, it is suitable to explore certain correlations between the measures and, finally, to learn whether the complexity indices capture structural information differently or similarly.

As a conclusive remark, we emphasize that the presented information-theoretic methods to analyze complex networks bear a considerable potential. Our study aimed to get a better understanding towards the problem of characterizing chemical graphs using information-theoretic complexity measures. In this paper, we put the emphasis on such measures which have already been applied in the context of mathematical chemistry and drug design. We think that our results can help to apply the measures to more complex network classes and to interpret the results more adequately than before.

In the future, we want to extend our measures for determining the structural complexity of weighted chemical graphs (i.e., incorporating atom and bond types) and test their ability to tackle QSAR/QSPR problems. Further, we would like to test novel information indices by combining existing ones and evaluate their discrimination power. Moreover, an interesting task would be to classify molecules by using this approach and to apply it to special problems in drug design.
